# Protocol to isolate and quantify large aging neutrophil-derived vesicles

**DOI:** 10.1016/j.xpro.2025.103886

**Published:** 2025-06-10

**Authors:** Alan Y. Hsu, Qingxiang Huang, Sizhou Feng, Hongbo R. Luo

**Affiliations:** 1 Department of Pathology, PhD Program in Immunology, Harvard Medical School, Mass General Brigham, Dana-Farber/Harvard Cancer Center, Boston, MA 02115, USA; 2 State Key Laboratory of Experimental Hematology, National Clinical Research Center for Blood Diseases, Institute of Hematology & Blood Diseases Hospital, Chinese Academy of Medical Sciences & Peking Union Medical College, Tianjin 300020, China

**Keywords:** Cell Biology, Cell isolation, Flow Cytometry, Immunology

## Abstract

Large aging neutrophil-derived vesicles (LAND-Vs) are ∼1 μm ectosomes released by aging neutrophils. Here, we present a protocol to identify and purify murine LAND-Vs from *in vitro* neutrophil cultures and *ex vivo* sources, including bone marrow, blood, and bronchoalveolar lavage fluid (BALF), using centrifugation and fluorescence-activated cell sorting (FACS). We also describe steps for isolation and quantification of human LAND-Vs from isolated neutrophils and human blood.

For complete details on the use and execution of this protocol, please refer to Hsu et al.^1^

## Highlights

• Optimized FACS protocol to isolate LAND-Vs from neutrophils, blood, and BALF
• Isolated LAND-Vs are suitable for comprehensive downstream analyses and functional assays
• Detailed sample preparation and gating strategy for LAND-V identification

## Abstract (editor highlights)

Publisher’s note: Undertaking any experimental protocol requires adherence to local institutional guidelines for laboratory safety and ethics.

## Graphical abstract

**Figure:**
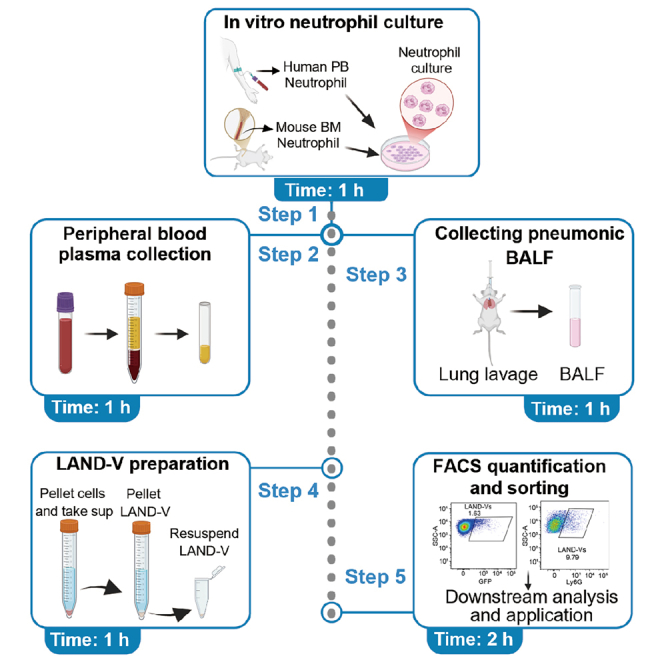

## Before you begin

To maximize experimental success, it is essential to maintain all samples, reagents, and materials on ice or at 4°C unless otherwise specified. Additionally, ensure that all necessary reagents, equipment, and workstations are prearranged and ready before initiating the protocol.

### Institutional permissions

#### Mice

All mouse experiments must comply with institutional and national guidelines. Obtain necessary institutional approvals before starting. Mouse studies were approved by the Institutional Animal Care and Use Committee at Brigham and Women’s Hospital. All animal experiments were conducted in accordance with the Animal Welfare Guidelines of the Brigham and Women’s Hospital with all procedures and monitoring approved. For experiments conducted at Brigham and Women’s Hospital, mice were housed under specific pathogen-free conditions, under a standard 12 h light cycle with ad libitum access to food and water, 21.7 C +/− 1.7C, 35%–70% +/− 5% humidity. All the procedures were approved under the Institutional Animal Care and Use Committee (IACUC) and operated under the supervision of the department of Center for Comparative Medicine (CCM). 8–10-week-old female C57BL/6J mice were purchased from the Jackson Laboratory (Bar Harbor, ME). B6.Cg-Tg(*Mrp8-Cre*,-EGFP)1Ilw/J, and *ROSA-26-mTmG* mice were inbreed and maintained in a pathogen-free system. *ROSA-26-mTmG* mice were crossed with B6.Cg-Tg(*Mrp8-Cre*,-EGFP)1Ilw/J mice to obtain mice in which neutrophils specifically express EGFP while all other cells express tdTomato. For peripheral blood and pneumonia studies, female mice of 8–10-week-old were used. For in vitro studies, mice of both sexes between 8–10-week-old were used.

#### Human samples

Ensure your lab has IRB approval or appropriate consent from donors before collecting human blood samples. All human neutrophils were isolated from apheresis-derived buffy coats from healthy donors as previously described.^1^ Peripheral blood samples were collected in EDTA tubes from patients admitted to VA Boston Healthcare System as part of their routine clinical Complete Blood Count (CBC) workups. The remaining blood samples were retrieved from the lab and used for research. This study has been approved by the Institutional Review Board of VA Boston Healthcare System. Donor information was redacted.

### Prepare the following

1. Purified neutrophils (Mouse bone marrow and human peripheral blood neutrophils are isolated as previously described using density gradient centrifugation or commercial kits^1^^,^^2^) Neutrophils should be cultured at a density of 2 × 10^6^ cells/mL in RPMI 1640 medium supplemented with 10% heat-inactivated fetal bovine serum and penicillin-streptomycin. Neutrophils are incubated at 37°C in a 5% CO_2_ incubator using non-tissue culture-treated plates.
2. Prepare sorting buffer and bronchoalveolar lavage buffer.
3. Set cell incubator to 37°C with 5% CO_2_.
4. Cool a centrifuge for 5 mL, 15 mL, or 50 mL tubes to 4°C which is able to run at 3000 × *g*.

## Key resources table

**Table:** 

REAGENT or RESOURCE	SOURCE	IDENTIFIER
**Antibodies**		

Brilliant Violet 421 anti-mouse Ly6G antibody (stock 0.2 mg/mL; final 2 μg/mL)	BioLegend	Cat#127627; RRID: AB_10897944
APC anti-mouse CD42d antibody (stock 0.2 mg/mL; final 2 μg/mL)	BioLegend	Cat#148505; RRID: AB_2564601
Brilliant Violet 421 rat IgG2a, κ isotype Ctrl antibody (stock 0.2 mg/mL; final 2 μg/mL)	BioLegend	Cat#400535; RRID: AB10933427
APC Armenian hamster IgG isotype Ctrl antibody (stock 0.2 mg/mL; final 2 μg/mL)	BioLegend	Cat#400911; RRID: AB_2905474
FITC anti-human CD66b antibody (stock 0.2 mg/mL; final 4 μg/mL)	BioLegend	Cat#305103; RRID: AB_314495
PE anti-human CD41 antibody (stock 0.2 mg/mL; final 4 μg/mL)	BioLegend	Cat#303706; RRID: AB_314376
FITC mouse IgM, κ isotype Ctrl antibody (stock 0.5 mg/mL; final 4 μg/mL)	BioLegend	Cat#401605; RRID: AB_389346
PE mouse IgG1, κ isotype Ctrl antibody (stock 0.2 mg/mL; final 4 μg/mL)	BioLegend	Cat#400112; RRID: AB_2847829
TruStain FcX (anti-mouse 16/32) antibody (stock 0.5 mg/mL; final 10 μg/mL)	BioLegend	Cat#101319; RRID: AB_1574973
FcR blocking reagent, human (stock 0.5 mg/mL; final 10 μg/mL)	Miltenyi	Cat#130-059-901; RRID: AB_2892112
Biotin anti-mouse CD41 antibody (stock 0.5 mg/mL; final 50 μg/mL)	BioLegend	Cat#133930; RRID: AB_2572133
Biotin anti-mouse TER-119/erythroid cells antibody (stock 0.5 mg/mL; final 50 μg/mL)	BioLegend	Cat#116203; RRID: AB_313704

**Bacterial and virus strains**		

*Staphylococcus aureus*	ATCC	Cat#10390

**Chemicals, peptides, and recombinant proteins**		

EDTA	Thermo Fisher Scientific	Cat#17892
Dextrose	Gibco	Cat#15023021
LPS	Sigma-Aldrich	Cat#L2630

**Critical commercial assays**		

Quick Start Bradford protein assay kit	Bio-Rad	Cat#5000201EDU
CellEvent caspase-3/7 red	Invitrogen	Cat#C10430
MitoSpy Green	BioLegend	Cat#424805
EasySep mouse neutrophil enrichment kit	STEMCELL Technologies	Cat#19762
EasySep direct human neutrophil isolation kit	STEMCELL Technologies	Cat#19666

**Experimental models: Organisms/strains**		

C57BL/6J; age 8-10 weeks; males and females	The Jackson laboratory	RRID: IMSR_JAX: 000664
B6.Cg-Tg(*Mrp8-Cre*,-EGFP)1Ilw/J; age 8–10 weeks; males and females	The Jackson laboratory	RRID: IMSR_JAX: 021614
B6.129(Cg)-*Gt(ROSA)26Sor*^*tm4(ACTB-tdTomato,-EGFP)Luo*^/J; age 8–10 weeks; males and females	The Jackson laboratory	RRID: IMSR_JAX: 007676

**Software and algorithms**		

Fiji/ImageJ	NIH	https://imagej.net/Fiji/Downloads; RRID: SCR_003070
FlowJo v.10.4.1	FlowJo	https://www.flowjo.com/; RRID: SCR_008520

**Other**		

PBS	Thermo Fisher Scientific	Cat#14190144
Fetal bovine serum	Gibco	Cat#16000044
RPMI 1640	Gibco	Cat#C11875500BT
BAMBANKER Direct	Bulldog Bio	Cat#BBD01
Penicillin streptomycin	Thermo Fisher Scientific	Cat#15070063
50 mL sterile centrifuge tube	Thermo Fisher Scientific	Cat#0644320
CountBright Plus absolute counting beads	Thermo Fisher Scientific	Cat#C36995
1 mL syringe	BD	Cat#300841
Goldenrod small animal lancet	Texas Scientific Instruments	N/A
Heparin-coated tube	BD Vacutainer	Cat#367886
EDTA-coated tube	BD Vacutainer	Cat#366643
1.5 mL microcentrifuge tube	Thermo Fisher Scientific	Cat#69715
5 mL polystyrene round-bottom tube	Falcon	Cat#352054
i.v. catheter	BD	Cat#381112

## Materials and equipment

**Table:** Bronchoalveolar lavage buffer

Reagent	Final concentration	Amount
PBS	N/A	500 mL
Heparin (1000 U/ml)	1 U/ml	0.5 mL
Dextrose	0.1%	0.5 g

EDTA can be added to 100 μM to prevent aggregation during collection if the pneumonia model used leads to massive cell infiltration. Bronchoalveolar lavage buffer can be prepared beforehand and stored at 4°C for up to 2 months.

**Table:** Sorting buffer

Reagent	Final concentration	Amount
PBS	N/A	100 mL
Heat-inactivated FBS	0.5%	500 μL

Sorting buffer can be prepared beforehand and stored at 4°C for up to 2 months.

## Step-by-step method details

### Isolation of LAND-V from mouse neutrophil *in vitro* culture with subsequent detection and sorting via flow cytometry

**Timing: 1–2 h**

**Figure 1:**
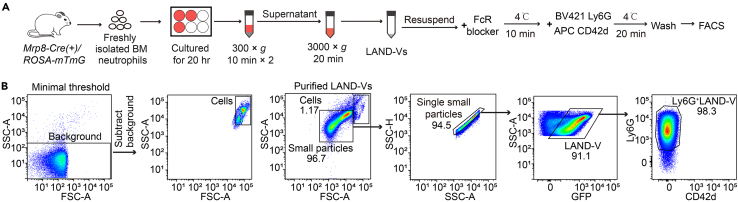
Identification of bone marrow derived LAND-Vs (A) Flowchart of LAND-V purification from isolated mouse bone marrow neutrophils and subsequent detection via flow cytometry. (B) Gating strategy to identify LAND-Vs via GFP expression and/or Ly6G staining. Gating was determined by isotype control samples respective of each channel.

The primary objective of this step is to purify and quantify LAND-V from mouse neutrophils from in vitro culture for subsequent characterization, quantification and functional studies (Figure 1A). *MRP8-Cre(+)/ROSA-mTmG* mice which express membrane localizing GFP^3^ specifically on neutrophils are used but WT mice can be also used and LAND-Vs identified by Ly6G staining.

1. Collect the 20 h cultured neutrophils into a 50 mL sterile centrifuge tubes.
2. Centrifuge at 300 × *g* for 10 min at 4°C. Then gently, collect the supernatant and discard the pellet to remove intact neutrophils and large debris. Repeat this step to ensure full removal of cells and debris.
3. Centrifuge the supernatant from step 2 at 3000 × *g* for 20 min, 4°C, collect the pellet and resuspend in 20 mL of PBS, followed by centrifugation at 3000 × *g* for 20 min, 4°C.

***Note:*** This sequential centrifugation strategy is adapted from other protocols used to for apoptotic bodies^4^ or migrasomes^5^ and adjusted based on the predicted size for LAND-Vs (∼1 μm).

4. Re-suspend the pellet in 500 μL of PBS for each 50 million starting neutrophils.

***Note:*** For functional assays, the suspension can be used directly for further studies. LAND-Vs can be stored in 4°C for 8 h. Longer periods of storage are not recommended.

5. Pipette 10 μL of the sample into a 5 mL round-bottom tube and add 100 μL of sorting buffer.

***Note:*** Sorting buffer can be prepared beforehand and stored at 4°C for up to 2 months.

6. Add 2 μL of TruStain FcX reagent and incubate at 4°C for 10 min to block non-specific binding of immunoglobulin.
7. Add 1 μL of BV421-conjugated Ly6G (200 ng) and 1 μL of APC-conjugated CD42d antibodies (200 ng). Include a separate tube with respective isotype controls and incubate at 4°C for 15 min in the dark.

***Note:*** All antibodies were used without prior dilution and applied in excess to ensure complete staining of the sample. Alternative fluorophore conjugates may be used, depending on the available flow cytometer configuration.

8. Wash the sample twice with 2 mL of PBS by centrifuging at 3000 × *g* for 5 min, 4°C each time.
9. Re-suspend the pellet in 200 μL PBS and add 10 μL CountBright Plus Absolute Counting Beads. Samples are now ready to perform flow cytometry analysis.
10. An example of the gating strategy is presented in Figure 1B.

**CRITICAL:** This analysis should be performed within the next 2 h, fixation is not recommended. AttuneNxT flow cytometer settings were: Forward Scatter (FSC) at 100, Side Scatter (SSC) at 250 with a 2500 FSC threshold applied, APC PMT gain at 400, FITC PMT gain 350, and Brilliant Violet 421 (BV421) PMT gain at 350. During acquisition, maintain a flow rate below 10,000 events per second. Note that optimal settings may vary between flow cytometers; therefore, it is recommended to perform a series of control experiments to establish a reliable working template.

***Note:*** LAND-Vs are identified after excluding background signal and regions where cells would appear, ensuring purity based on FSC-A (Forward Scatter Area) and SSC-A (Side Scatter Area) distribution (Figure 1B). Using a logarithmic scale for FSC-A and SSC-A instead of a linear scale improves the discrimination of LAND-Vs from background noise and larger cells. Additionally, filtering the sorting buffer through a 0.1 μm membrane filter helps reduce background noise in the SSC/FSC channels and prevents contamination from the buffer itself.

11. Discard doublets using SSC-A and SSC-H (Figure 1B).
12. LAND-Vs derived from GFP-expressing neutrophils are defined as a GFP-positive population (>90%). Within this population, over 95% of GFP^+^ LAND-Vs also express Ly6G, indicating that Ly6G can serve as a marker for LAND-Vs (Figure 1B). Based on this, we designate Ly6G as a reliable marker for identifying LAND-Vs from in vitro neutrophil culture.

***Note:*** If GFP mice are not readily accessible, especially since genetic knockout mice typically do not express GFP. One can directly stain with Ly6G which is a marker for murine neutrophils^6^ as a surrogate marker to represent LAND-Vs.

13. Perform LAND-V quantification using the following formula: indicated collected LAND-Vs count /collected beads count × total beads added.
14. If ultrapure LAND-Vs is required, you can sort LAND-V based on the gating strategy defined in Step 12, which can be used for downstream analysis.

***Note:*** Given their size, most FACS sorters can detect and sort LAND-Vs without the need for a specialized SSC filter. The gating strategy for isolating total LAND-Vs is outlined in Figure 1B. To preserve vesicle integrity, it is recommended to include protease inhibitors in the collection tubes and to perform sorting at 4°C. Use a sorting nozzle ≥70 μm and maintain the slowest possible flow rate that still allows for the collection of approximately 2,000 LAND-Vs per second, with droplet efficiency exceeding 85%.

***Note:*** If human LAND-Vs isolated from ex vivo purified neutrophils are needed, it is recommended to collect them after 40 h of culture, as human neutrophils have a longer half-life than their murine counterparts.^7^^,^^8^ After the 3000 × *g* spin, collect the pellet and stain with anti-human CD41 and anti-human CD66b with respective isotype controls to identify LAND-Vs as we previously shown,^1^ along with other markers one may desire to assess and follow steps 9–14. Purification of human neutrophils from peripheral blood was done as previously described.^9^

***Note:*** Our FACS analysis of LAND-Vs generally aligns with MIFlowCyt-EV guidelines^10^; however, some criteria could not be met due to the primary isolation of human and mouse LAND-Vs and the inherent variability in their marker expression across individual biological samples.

### Identification of LAND-V in mouse peripheral blood by flow cytometry

**Timing: 1–2 h**

**Figure 2:**
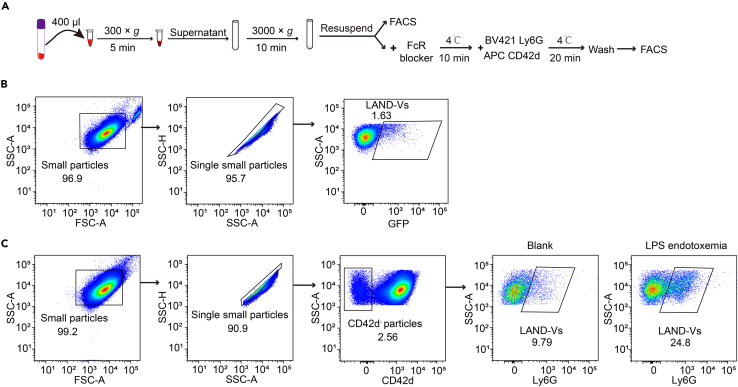
Identification of LAND-V from murine plasma (A) Flowchart of LAND-V purification from mouse peripheral blood and subsequent detection via flow cytometry. (B) Gating strategy to identify LAND-Vs via GFP expression. (C) Gating strategy to identify LAND-Vs via platelet (CD42d) exclusion and Ly6G staining. Gating was determined by isotype control samples respective of each channel. The right panel shows the increase of LAND-Vs in the plasma in response to endotoxemia challenge.

This section describes the methods for identifying LAND-V in peripheral blood (PB), while also showing the change in LAND-V numbers in the PB during infection (Figure 2A).

15. Collect peripheral blood of *MRP8-Cre (+)/ROSA-mTmG* mice via submandibular venipuncture using a 4–5 mm lancet and collect the blood into a vacutainer tube containing EDTA or heparin to prevent coagulation.

***Note:*** Additional EDTA (2 mM final concentration) or heparin (100 U/ml final concentration) may be added to ensure minimal coagulation which will not affect LAND-Vs during the duration of collection.

16. Pipette 400 μL of anticoagulated blood into a 1.5 mL microcentrifuge tube.
17. Centrifuge at 300 × *g* for 5 min, RT. Collect the supernatant into 5 mL round-bottom tube carefully and discard the pellet to remove cells.

***Note:*** If the supernatant still appears red due to residual red blood cells, centrifuge at 100 × *g* for 5 min, RT to remove RBC and transfer supernatant into a new 5 mL tube.

**CRITICAL:** RBC lysis buffer is not recommended as the lysed RBC debris will interfere with LAND-V detection and purity.

18. Centrifuge supernatant at 3000 × *g* for 10 min, 4°C.
19. Resuspend the pellet in 300 μL PBS and perform flow cytometry analysis.
20. Gating strategy is provided in Figure 2B.
21. Following doublet depletion, the GFP-positive particles are designated as LAND-Vs and can be sorted or quantified for downstream applications.
22. If the neutrophils from the mice used does not express GFP, collect LAND-V samples from mouse peripheral blood according to steps 15–18, resuspend in 100 μL PBS and then block Fc receptors with TruStain FcX and stain for Ly6G and CD42d. Include a separate tube with respective isotype controls as described in steps 6–9.
23. Gating strategy is provided in Figure 2C.
24. Discard doublets using SSC-A and SSC-H (Figure 2C).
25. Exclude platelet vesicles and enrich for LAND-Vs by gating out the CD42d-positive population.
26. Gate Ly6G-positive particles and define them as LAND-Vs.
27. It is not recommended to freeze LAND-Vs as there could be disruption to the vesicles, but if needed, mix the resuspended LAND-Vs 1:1 with BamBanker Direct (Bulldog bio#BBD01) and store in −80^o^C for up to 6 months.

***Note:*** The provided example shows LAND-V regulation during an LPS endotoxemia model, where LPS was administered intraperitoneally at a dose of 2.5 mg/kg and collected at 6 hpi (Figure 2C).^11^ This model is used to assess the kinetics of LAND-V in the circulation in response to acute infection.

### Identification and purification of LAND-V in bronchoalveolar lavage fluid by fluorescence-activated cell sorting

**Timing: 2–3 h**

The purpose of this section is to identify LAND-V in bronchoalveolar lavage fluid (BALF) and evaluate its changes under steady-state conditions or in the context of pulmonary diseases. Furthermore, the identified BALF LAND-Vs can be sorted for subsequent experimental applications.

**Figure 3:**
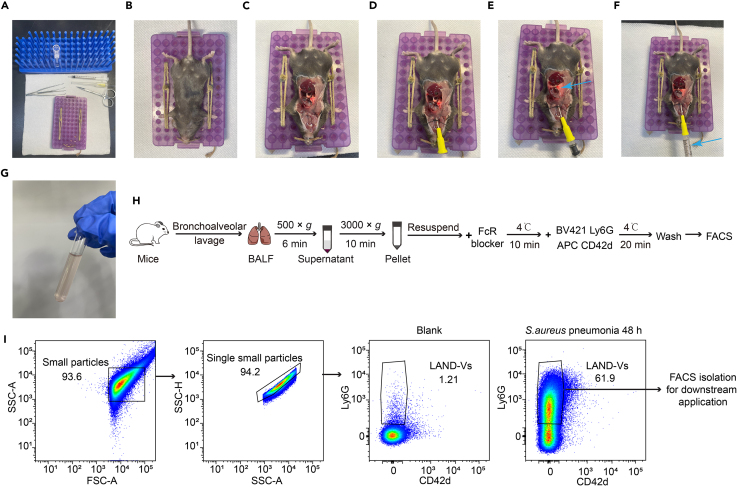
Lavaging of the pneumonic lung to isolate LAND-Vs from the alveolar space (A) Materials needed: (1) cold bronchoalveolar lavage buffer, (2) forceps, (3) surgery scissors, (4) dissection table with restrainer (see image), (5) 1 mL syringe, (6) 24G catheter needle, (7) suture thread, (7) 5 mL tube, and (8) 70% ethanol. (B) Euthanize the mice according to the approved procedure (CO_2_ euthanasia with subsequent cervical dislocation) and secure the mice on the restrainer as shown in the figure. (C) Perform vertical incision to the thoracic skin and muscle layers and cut open the rib cage, exposing the lung and trachea. Take caution not to puncture the lung. (D) Insert the catheter into the trachea ∼1 cm and tie a double surgeon’s knot with the suture securing the catheter in the trachea. Remove the needle. (E and F) Slowly inject 800 μL of cold bronchoalveolar lavage buffer into the lungs and observe the lungs slowly expand with no leakage of fluid (blue arrow). Aspirate the buffer back into the syringe gently to prevent the formation of vacuum and damage of lung tissue. Repeat this step three times. (G) Collect the BALF in a 5 mL tube containing 3.0–3.2 mL of BALF. (H) Flowchart of BALF LAND-V detection via flow cytometry. (I) Gating strategy to identify BALF LAND-Vs via Ly6G staining. Gating was determined by isotype control samples respective of each channel.

***Note:*** Bronchoalveolar lavage buffer can be prepared beforehand and stored at 4°C for up to 2 months.

28. Prepare the procedure space (Figure 3A). You will need:
  a. 5 mL polystyrene round-bottom tube with 3.2 mL cold bronchoalveolar lavage buffer to collect the BALF.
  b. Surgery scissors and forceps for the dissection.
  c. Dissection surface.
  d. 24G i.v. catheter (for lavage).
  e. 1 mL syringe.
  f. Bronchoalveolar lavage buffer.

29. Euthanize mice following your institutional animal policy. We use a CO_2_ chamber in combination with cervical dislocation.
30. Position and restrain the mouse (Figure 3B).
  a. Place mouse in a supine position on the stabilizing device.
  b. Immobilize the animal by securing its head to fully expose the neck.
  c. Secure the front and back paws, then spray the torso with 70% ethanol if sterility is needed to reduce fur contamination and disinfect the incision site.
31. Make a midline abdominal incision along the anterior midline of the mouse, extending from the cervix to the lower edge of the ribs. Use forceps to lift the xiphoid process of the sternum and remove the ribs to open the thoracic cavity, ensuring not to damage the lungs (Figure 3C).

***Note:*** When opening the thoracic cavity, angle the scissors upward to avoid damaging the lung tissue.

32. Gently dissect the anterior cervical fascia and muscles using curved forceps to expose the white trachea. Perform blunt dissection around the trachea and thread a suture beneath it.
33. Insert an i.v. catheter into the upper half of the trachea, securing it with the pre-placed suture. Remove the needle core and attach a 1 mL syringe loaded with 800 μL of cold PBS to the cannula (Figure 3D).
34. Inject the buffer into the lungs through the cannula, observing the steady inflation of the lungs (Figure 3E). Aspirate the liquid back into the syringe twice. The expected recovery is approximately 500–700 μL (Figure 3F).
35. Perform the lavage procedure three times more to ensure thorough rinsing of the lungs (Figure 3G).
36. Collect the BALF, Centrifuge at 500 × *g* for 5 min, RT. Collect the supernatant and discard the pellet to remove cells and large debris.
37. Centrifuge the supernatant (from step 36) at 3000 × *g* for 10 min, 4°C. Re-suspend the pellet in 100 μL PBS (Figure 3H).
38. Block Fc receptors with TruStain FcX and stain for Ly6G and CD42d. Include a separate tube with respective isotype controls following steps 6–9.
39. Gating strategy is provided in Figure 3I.
40. Discard doublets using SSC-A and SSC-H (Figure 3I).
41. Gate Ly6G-positive particles and define them as LAND-Vs.

***Note:*** The number of LAND-Vs in BALF varies based on the type of pneumonic inducer and the time of collection. For purification in subsequent studies, optimize conditions to maximize yield during cell sorting.

### Identification and quantification of LAND-Vs from human plasma by flow cytometry

**Timing: 1–2 h**

42. Collect 1 mL of human venous blood into a vacutainer tube containing EDTA or heparin.
43. Directly spin the whole blood at 300 × *g* (5 min, RT) and carefully transfer 400 μL of suspending plasma into a new 5 mL tube without disrupting the sedimented cells.

***Note:*** If the supernatant still appears red due to residual red blood cells, repeat step 43.

44. Spin the plasma at 3000 × *g* (5 min, 4°C) to pellet LAND-Vs and other large vesicles.
45. Remove the supernatant and resuspend the pellet in 100 μL Sorting buffer.
46. Add 2 μL of human FcR blocking reagent and incubate at 4°C for 10 min to block non-specific binding of immunoglobulin.
47. Add 2 μL anti-human CD41 (400 ng) and anti-human CD66b (400 ng) and a separate tube with respective isotype controls to identify LAND-Vs as we previously shown^1^ along with any other markers desired and incubate at 4°C for 15 min in the dark.

***Note:*** All antibodies were used without prior dilution and applied in excess to ensure complete staining of the sample. Alternative fluorophore conjugates may be used, depending on the available flow cytometer configuration.

48. Wash the sample twice with 2 mL of sorting buffer by centrifuging at 3000 × *g* (5 min, 4°C) each time.
49. Re-suspend the pellet in 200 μL PBS and add 10 μL CountBright Plus Absolute Counting Beads. Samples are now ready to perform flow cytometry analysis. This analysis should be performed within the next 2 h, fixation is not recommended.
50. The gating strategy is as previously described.^1^ LAND-Vs were identified after excluding background signal and regions where cells would appear, ensuring purity based on FSC-A and SSC-A distribution, and excluding platelet EVs via CD41 expression and utilizing CD66b as a surrogate marker for human LAND-Vs. Calculation of LAND-Vs is as described in step 13.

## Expected outcomes

Based on FACS quantification with counting beads the routine yield from 1 × 10^6^ *in vitro* cultured mouse bone marrow neutrophils after 20 h culture, is 3–5 × 10^6^ LAND-Vs, with a protein content of 8–12 μg, as quantified by BCA assay. From 400 μL of peripheral blood collected from 8–12-week-old female mice, the number of LAND-Vs identified is approximately ∼4 × 10^4^. In a mouse pneumonia model induced by 50 million *S. aureus*, ∼5 × 10^6^ LAND-Vs can be isolated from the bronchoalveolar lavage fluid (BALF) at 48 hpi. LAND-Vs can then be quantified or characterized as previously shown.^1^ For LAND-Vs in the peripheral blood of a healthy human, 100 μL of plasma is expected to contain 1–3 × 10^4^ LAND-Vs. Notably, there is an approximate 40% loss of purified LAND-Vs following a single freeze-thaw cycle even in the presence of cryopreservatives, as well as LAND-Vs isolated from frozen plasma.

## Limitations

Though we have previously characterized a panel of neutrophil expressing markers on LAND-Vs, and identified enriched proteins compared to neutrophils, we still rely on FACS validation to confirm expression of a given protein on the surface of LAND-Vs. When utilizing a GFP neutrophil mice strain we can detect LAND-Vs *in vivo*. However, since other extracellular vesicles share these properties, they can’t be used as unique defining features—aside from size or ex vivo staining. Our purification method with differential centrifugation steps may also contain low percentages of apoptotic bodies, smaller microvesicles and possibly neutrophil granules. Utilizing GFP or Ly6G expression and size is the only current method of ensuring LAND-V quantification, though it is an underestimation of total LAND-V abundance. Additionally, in vivo quantification of LAND-V is highly dependent upon collection method and stimulation used, where neutrophil function and recruitment dynamics could vary. We currently lack a reliable method to collect endogenous human LAND-Vs outside of peripheral blood. However, our previous findings indicate that CD66^+^ DAPI^−^ particles (∼1 μm) can be detected and was enriched in pneumonic lung sections compared to healthy controls.^1^ Therefore, tissue section imaging may serve as an alternative approach to detect and partially quantify LAND-Vs in human tissue.

## Troubleshooting

### Problem 1

LAND-Vs derived from BM neutrophil culture sometimes may be contaminated with other type of vesicles. Step 12.

### Potential solution

The purity of LAND-Vs primarily depends on the purity of the isolated neutrophils. Therefore, ensuring high-purity neutrophils is essential. If the Percoll gradient centrifugation yields a purity below 90%, further purification can be achieved using a negative selection commercial purification kit. Alternatively, a negative selection kit can be used directly without prior purification though it may isolate neutrophil progenitors as well leading to skewed LAND-V yield.

### Problem 2

Murine LAND-Vs represent less than 5% of total events in 3000 × *g* pellet obtained from plasma, while LAND-Vs can also aggregate with other vesicles. Steps 23–26.

### Potential solution

To obtain an enriched LAND-V prep, after the 3000 × *g* centrifugation, resuspend the preparation in 100 μL PBS and subject it to negative selection using an EasySep Mouse Neutrophil Enrichment Kit (Stemcell#19762) with addition of 10 μL of biotin conjugated anti-CD41 (Biolegend#133930) and anti-Ter119 (Biolegend#116203) along with the neutrophil enrichment cocktail. This method significantly enriches LAND-Vs but the yield may be lost due to the removal of aggregated vesicles. To decrease vesicle aggregation, 1% BSA (0.1 μm filtered) can be added into all buffers but may introduce noise if the downstream mass spectrometry is desired.^12^

### Problem 3

Difficulty in distinguishing LAND-Vs with other neutrophil derived vesicles of similar size (such as apoptotic bodies).

### Potential solution

In our initial report we show that LAND-Vs are a distinct type of vesicle, but cannot rule out minimal contaminating vesicles. We have identified that 40%–60% of LAND-Vs express CD55 which was not expressed on apoptotic bodies.^1^ Thus, to definitively rule out the presence of apoptotic bodies, CD55 can be used as a surrogate marker for LAND-Vs. Additionally, to remove apoptotic bodies, cleaved caspase 3/7 or mitochondria (MitoSpy) staining can be used as LAND-Vs are negative for these markers.

## Resource availability

### Lead contact

Further information and requests for resources and reagents should be directed to, and will be fulfilled by the lead contact, Hongbo R. Luo (hrluo@bwh.harvard.edu).

### Technical contact

Questions about the technical specifics of performing the protocol should be directed to and will be answered by the technical contact, Alan Y. Hsu (ahsu7@bwh.harvard.edu).

### Materials availability

This study did not generate new unique resources, mice, or reagents. Further information on materials, datasets, and protocols should be directed to and will be fulfilled by the lead contact, Hongbo R. Luo (hrluo@bwh.harvard.edu).

### Data and code availability

This study did not generate new unique data or code resources.

## Acknowledgments

This work was supported by 10.13039/100000002National Institutes of Health grants 1R01AI142642, 1R01AI145274, 1R01AI141386, R01HL092020, and P01HL158688 to H.R.L. A.Y.H. was supported by 10.13039/100000002NIH training grant T32HL066987 and the Cotran-Gimbrone Research Award. S.F. is supported by the 10.13039/501100005150Chinese Academy of Medical Sciences (CAMS) Innovation Fund for Medical Sciences (grant number 2021-I2M-1-017) and the 10.13039/100014717National Natural Science Foundation of China (82470208).

## Author contributions

A.Y.H. and Q.H. performed experiments. A.Y.H. designed and supervised the protocol. H.R.L. designed and supervised the study. S.F. provided resources. A.Y.H., Q.H., and H.R.L. wrote the manuscript, which was edited by all authors.

## Declaration of interests

The authors declare no competing interests.
